# Increased Efficiency of Solar Cells Protected by Hydrophobic and Hydrophilic Anti-Reflecting Nanostructured Glasses

**DOI:** 10.3390/nano7120437

**Published:** 2017-12-14

**Authors:** Estela Baquedano, Lorena Torné, Pablo Caño, Pablo A. Postigo

**Affiliations:** 1 Instituto de Micro y Nanotecnología, CSIC, 28760 Tres Cantos, Madrid, Spain; estela.baquedano@csic.es (E.B.); lorena.torne@csic.es (L.T.); 2 IES-Instituto de Energía Solar, Universidad Politécnica de Madrid, 28040 Madrid, Spain; pablo.cano@ies-def.upm.es

**Keywords:** glass, nanostructuration, nanolithography, plasma etching, optical properties, solar cells, hydrophobic, hydrophilic

## Abstract

We investigated the fabrication of large-area (cm^2^) nanostructured glasses for solar cell modules with hydrophobic and hydrophilic properties using soft lithography and colloidal lithography. Both of these techniques entail low-cost and ease of nanofabrication. We explored the use of simple 1D and 2D nanopatterns (nanowires and nanocones) and the effect of introducing disorder in the nanostructures. We observed an increase in the transmitted light for ordered nanostructures with a maximum value of 99% for wavelengths >600 nm when ordered nanocones are fabricated on the two sides of the solar glass. They produced an increment in the efficiency of the packaged solar cell with respect to the glass without nanostructures. On the one hand, the wettability properties showed that the ordering of the nanostructures improved the hydrophobicity of the solar glasses and increased their self-cleaning capacity. On the other hand, the disordered nanostructures improved the hydrophilic properties of solar glasses, increasing their anti-fogging capacity. The results show that by selecting the appropriate nanopattern, the wettability properties (hydrophobic or hydrophilic) can be easily improved without decreasing the efficiency of the solar cell underneath.

## 1. Introduction

Solar energy is often seen as the most promising form of renewable energy, due to the amount of solar irradiation available for its generation [1,2]. High efficiency solar cells require high absorption of incident sunlight. In commercial mono and multi-crystalline Si cells based on solar cell wafers, absorption is routinely improved by surface texturing, typically with pyramid shapes of a few micrometers in size. This texturing increases the light scattering in the cell by an anti-reflective coating of silicon nitride [3,4]. This approach is not practical for thin film silicon solar cells, where the thickness of the absorbent layer is in the order of 1–3 μm and can be even less. In these cells, the absorption is improved by engineering the surface of the material used as the substrate or solar glass [5]. This process improves both the light scattering in the absorbent thin layer and the anti-reflective effect in the interfaces over a wide range of wavelengths and incidence angles [6,7]. The anti-reflective properties originate from a gradual change in the effective refractive index in textures with texture sizes where the smallest characteristic corresponds approximately to the wavelength of incident light [5,8]. 

The cover glass constitutes approximately 25% of the silicon thin film (Si) modules [9] and approximately 10–15% of the crystalline Si modules [10]. Therefore, the improvement of the cover glass becomes essential to reduce costs. In this context, there are two means of acting on glass to reduce costs. The first would be the reduction of costs in the manufacture of glass and the second, an increase in the transmission of sunlight through the glass, since a 5% increase in solar transmittance could result in an improvement of up to 10% in the efficiency of the solar module [11]. Current solar glasses transmit about 90% of incident light, due to loss of absorption and reflection, unless coated with some coating. In this case, it is possible to achieve transmissions >98%. The regular coatings are typically SiO_2_, Si_2_N_3_ or MgF_2_ porous films [12]. However, these coatings do not usually have a high hydrophobicity that would give them the ability to self-clean, and therefore would require maintenance. Self-cleaning liners are a way to achieve reduced maintenance. One of the most frequently used liners to obtain self-cleaning surfaces is the TiO_2_ [13,14]. However, these coatings have a higher refractive index than the glass of the substrate that increases the reflectivity. The self-cleaning surface technology is often related to the lotus flower effect [15], where the contact angle of the water is key. In this context, two relevant low-cost and scalable techniques are spray coating (commercial products are already disposable such as SurfaShield G by NanoPhos S.A) and dip-coating [16]. In order to use self-cleaning coatings with a similar index of refraction, nanostructuration of the solar glass is presented as a good option to improve both the transmission of sunlight and the ability to self-clean (hydrophobic) or prevent fogging (hydrophilic). By altering the roughness of the surface at the nanoscale, it is possible to create hydrophilic surfaces [17,18] that prevent fog or to create hydrophobic surfaces [19,20] that have a self-cleaning effect. In addition, this nanostructuration of the glass surface can improve the anti-reflective properties of glass [21,22]. This improvement of the solar glasses would avoid the need for coatings, thus reducing the costs of the solar module and ultimately increasing the efficiency of the cell. Nevertheless, it is essential that the procedures used for nanostructuration can produce the nanopatterning in an easy way, in large areas (at least in the order of several cm^2^), and with low-cost.

The most common methods for the creation of textured interfaces for thin film solar cells utilize the deposition of boron doped textured zinc oxide (ZnO:B) by low pressure chemical vapor deposition (LPCVD), or chemical etching of ZnO (AZO) in thin films using a dilute hydrochloric acid solution (HCl:H_2_O) [23,24]. These surface-textured techniques generate sub-micrometric features and are both scalable and economically inexpensive, making them attractive options for a commercial application. However, these approaches provide limited control over the size of the feature and the geometry of the nanometric texture can often result in a surface texture that is not fully optimized for light retention. To this end, different light management strategies have been explored, including the introduction of plasmon nanoparticles [25,26], ordered photonic [27,28] and disordered [3,4,6,7] nanostructures. These studies show that ordered nanostructures can provide a significant enhancement to the absorption in thin-layer solar cells, although this finding is often limited to relatively narrow wavelength ranges [29]. It is also known that an improvement in broadband absorption requires photonic structures with a certain amount of disorder. For this reason, disordered interactions [30,31,32] which are inherently broadband scatter centers, are particularly promising. However, the description of a realistic shape of its topography is complicated, which makes studying and optimizing the light that can be caught, a complicated task. Furthermore, the operational efficiency and the lifetime of the photovoltaic module would be increased by either a self-cleaning or anti-fogging effect, as well as by the improvement in the anti-reflectivity of a surface specially designed to achieve high optical transmission.

In this work, we have used low-cost methods to make large-area rough glass interfaces by nanostructuration. In particular, we focused on ultra-thin solar glass, aiming to achieve the transmission limit with optimized roughness, produced by a simple, low-cost nanostructuration. To create the texture at the nanoscale with a controlled geometry, two techniques of manufacture were explored: soft lithography was used to produce high quality 1D and 2D ordered patterns and colloidal lithography was used to fabricate 2D disordered nanostructures. Both techniques are simple, non-expensive, and allow the fabrication of large areas. The results obtained showed an improvement in the transmission of light for ordered nanostructures with a maximum value of 99% when 2D ordered nanostructures were fabricated on both sides of the solar glass. The measurement of wetting properties showed that the ordered nanostructures improved the hydrophobicity and the self-cleaning properties, whereas the disordered nanostructures improved the hydrophilic properties, without the use of any chemical treatment or coating of the surface. Finally, a solar cell packaged under the differently fabricated nanostructured glasses (without any optical glue or thermo-adhesive) has been measured. The results showed that different wettability properties do not significantly alter the final efficiency of the solar cell and that if the right nanopattern is chosen, the efficiency can be increased with respect to the use of a non-patterned glass. 

## 2. Experimental Process

In the study on the influence of shape, size, and arrangement of the nanostructures in the transmission and the wettability of the solar glass, two different methods were followed. 

**Figure 1:**
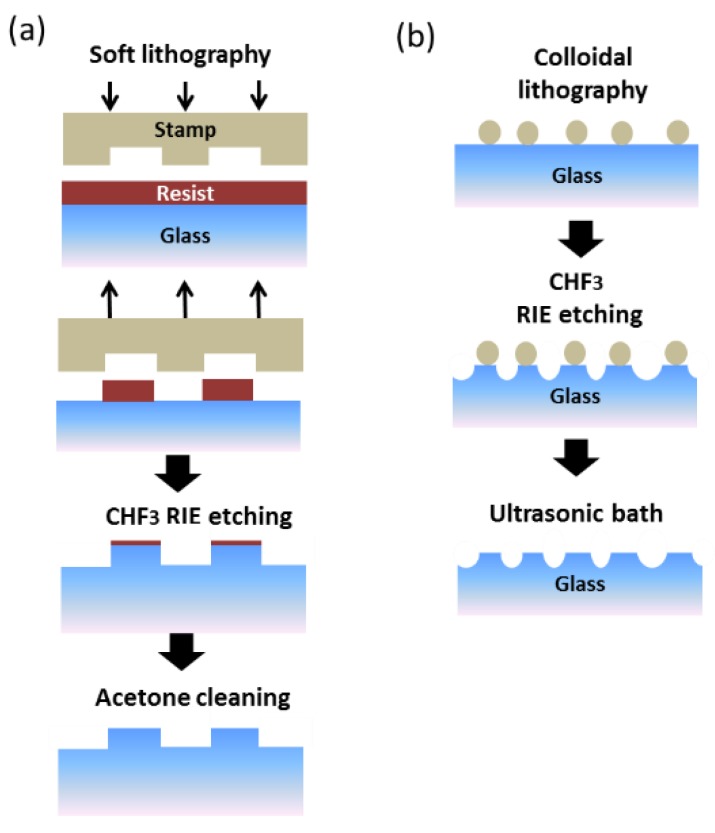
Fabrication processes. (**a**) Soft lithography for order samples (**b**) Colloidal lithography for disorder samples.

Figure 1a shows the fabrication process used for glasses with ordered patterns (1D-nanolines or -nanowires and 2D-nanocones). These glasses were patterned by soft lithography [33]. Elastomeric Polydimethylsiloxane (PDMS) stamps were fabricated using the following low-cost molds: (i) Compact Disc (CD) with a lineal grating of 1600 nm period and 600 nm linewidth; (ii) Digital Versatile Discs (DVD) with a lineal grating of 775 nm period and 400 nm linewidth; (iii) Blu-ray disc (BR) with a lineal grating of 325 nm period and 200 nm linewidth; (iv) master of silicon with pillars of 400 nm period and 170 nm diameter. This master was fabricated by laser interference lithography (LIL). A droplet of 5 μL of resist (5% PMMA 996 k in gamma-butyrolactone (GBL) was dropped over the glass (Menzel-Gläser cover slips 18 × 18 mm^2^, 130 μm to 160 μm thick) with a micropipette. The resist was then covered with the PDMS stamp with the desired pattern and was then pressed between two glasses to obtain a mask. The resist was cured under low-vacuum (desiccator) for 3 h [34]. All the procedures were extremely easy and cheap and permitted the fabrication of large areas, in our case limited to a size around 1.8 × 1.8 cm^2^ for easier handling during experimental measurements. Reactive Ion Etching (RIE) with CHF_3_ was used to transfer the pattern into the glass. Finally, the residual resist was washed off with acetone. Figure 1b shows the fabrication process used for glasses with disordered patterns. A commercial polystyrene (PS) nanoparticle solution was used to make the mask by colloidal lithography. The concentration of the solution was 2%, and the size distributions of the nanospheres were 124, 202, 500 and 1024 nm. RIE with CHF_3_ was used to transfer the pattern into the glass. Finally, the nanoparticles were washed off in an ultrasonic bath with milli-Q water.

## 3. Results and Discussion

### 3.1. Optical Characterization of Ordered 1D Nanostructures

**Figure 2:**
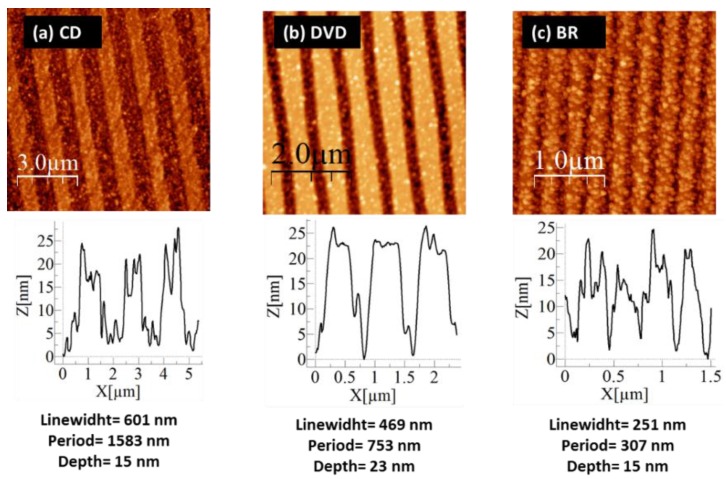
Atomic force microscope (AFM) images of ordered 1D nanostructures on glass. (**a**) Compact disc (CD) sample. (**b**) Digital versatile disc (DVD) sample. (**c**) Blu-ray disc (BR) sample.

Figure 2a–c show atomic force microscope (AFM) images for the fabricated samples. The periods obtained were 307, 753 and 1583 nm and were denominated BR, DVD and CD, respectively. The depths of the nanostructures were between 15 and 25 nm. 

**Figure 3:**
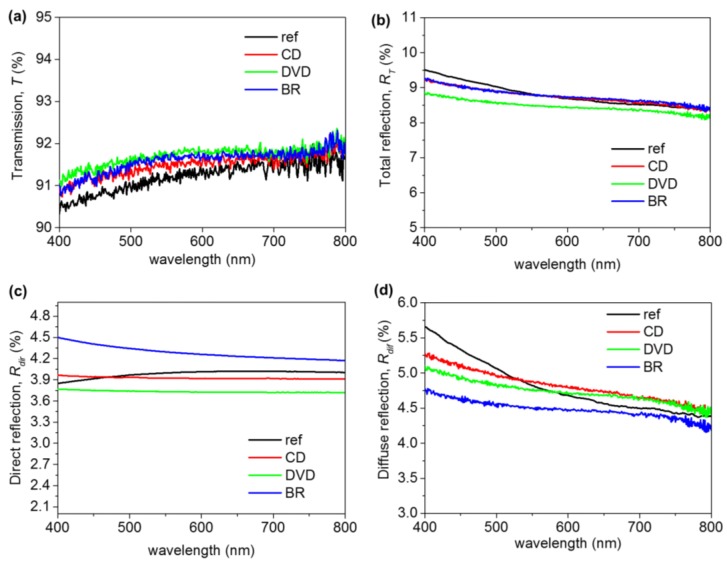
Optical measurements of ordered 1D nanostructures. (**a**) Transmission spectra (*T*). (**b**) Total reflection spectra (*R_T_*). (**c**) Direct reflection (*R_dir_*). (**d**) Diffuse reflection (*R_dif_*).

Optical measurements were realized using an integrating sphere (Perkin Elmer Lambda 950) with universal reflectance accessory (URA) that had a rectangular spot size of around 4 × 9 mm^2^. The measurements were carried out with an incidence of 8° and without any polarization filters. Figure 3 shows the optical spectra measured, compared to the same glass without nanostructures (labeled “ref”). Figure 3a shows the transmission (*T*) spectrum obtained for each nanostructured glass and for the reference sample. For all the cases, *T* smoothly increases towards the infrared region. It was also observed that for all of the nanostructured glasses, a slight improvement of the *T* was achieved with respect to the reference sample. This improvement was more remarkable at short wavelengths. The higher improvement in *T* (around 0.5%) was achieved for the DVD sample and also for the short wavelengths (400–600 nm). In any case, no clear influence of the period used for the fabrication of the 1D nanostructures was observed. Figure 3b shows the total reflection (*R_T_*) measured in the integrated sphere, represented as the sum of the direct and diffuse reflection. The spectra show a decrease of the *R_T_* with respect to the reference sample for all the nanostructured glasses at short wavelengths. This is in accordance with the increase of the *T* in the same wavelength range. However, towards longer wavelengths, only the *R_T_* of the DVD is smaller than in the reference sample. Figure 3c shows the direct reflection spectrum (*R_dir_*) being corrected for a thin glass according to Fresnel’s law [35]. The plot shows that *R_dir_* remains approximately constant for the visible spectrum. Only in the case of BR, was a slight decrease observed at short wavelengths. Figure 3d shows the diffuse reflection or light scattering (*R_dif_*) calculated as *R_dif_* = *R_T_* − *R_di_*_r_. A decrease in *R_dif_* was observed towards large wavelengths for all samples. Here, we can see more clearly that in the case of the BR sample, the scattering of the light was smaller than the reference sample for all the visible wavelengths. Meanwhile, in the cases of CD and DVD, the light scattering is higher for wavelengths longer than 530 nm and 575 nm respectively. This observation matches well with the fact that the BR sample has the nanostructures of a smaller size. As a partial conclusion, 1D ordered nanostructures (nanolines forming a grating) always improve *T,* although only rather slightly and without a clear dependence on the period used. The scattering effect (haze) that has been described as relevant in solar cell enhancement was moderated (around 5%).

### 3.2. Optical Characterization of Disordered 2D Nanostructures

**Figure 4:**
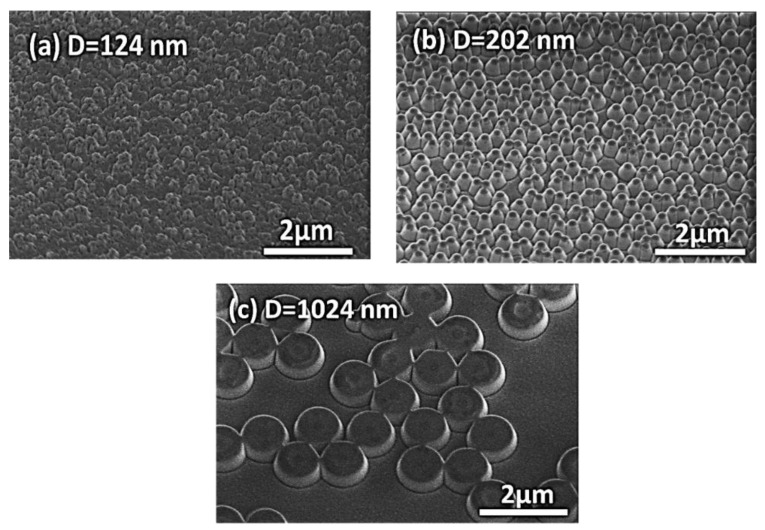
SEM images of disordered 2D nanostructures, tilt 30°. (**a**) D = 124 sample. (**b**) D = 202 sample. (**c**) D = 1024 sample.

Figure 4a–c show scanning electron microscope (SEM) images of the nanostructures obtained in the surfaces of the glass using PS nanoparticles with different diameters (D = 124, 202 and 1024 nm). The height of all the nanostructures was ≈200 nm.

**Figure 5:**
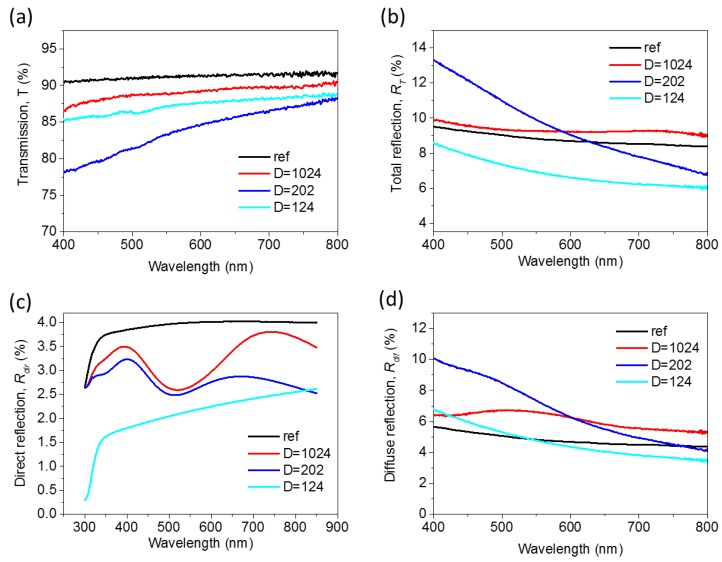
Optical measurements of disordered 2D nanostructures. (**a**) Transmission spectra (T). (**b**) Total reflection spectra (RT). (**c**) Direct reflection (*R_dir_*). (**d**) Diffuse reflection (*R_dif_*).

Figure 5a shows *T* versus the wavelength, for different diameters of the disordered 2D nanospheres used, as well as for the reference sample. Contrary to the case of the 1D nanolines, none of the samples improved the *T,* despite the large range of diameters used. The decrease in size induces an increase in the degree of disorder, which seems to be the main reason for the decrease of *T*. The direct reflection shown in Figure 5c has values around those that were observed for the 1D ordered nanolines. Nevertheless, the large diffuse reflection scattering shown in Figure 5d is more than double of that of the 1D ordered samples. We must note that there is a very small amount of absorption in the case of sample D = 1024 due to the presence of PS remains (as has been checked by AFM). This makes that the sum of *R_T_* (Figure 5b) and *T* is slightly below 100% but it does not alter the main conclusion of the study: disorder does not seem to enhance *T* but rather contributes largely to scattering and haze. Indeed, most of the samples in this batch looked slightly white to the eyes.

### 3.3. Optical Characterization of Ordered 2D Nanostructures

**Figure 6:**
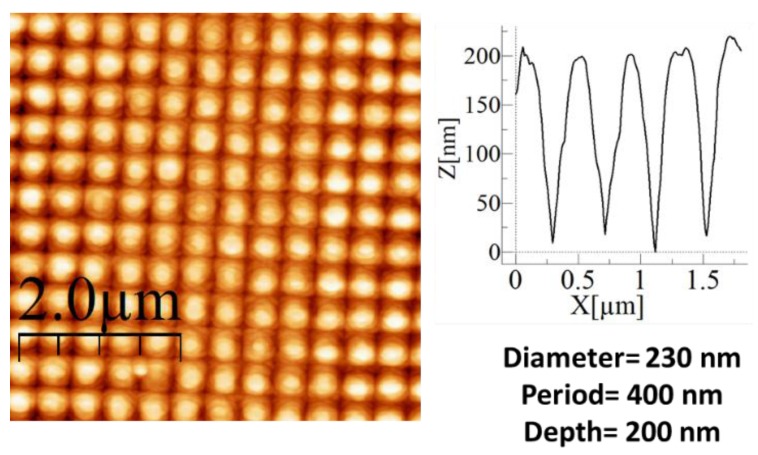
AFM image of ordered 2D nanostructures on glass.

Since the 1D ordered nanopatterning seemed to be adequate in providing an enhancement for *T*, we extend the ordering to 2D using nanocones. Nanocones or nanoneedles have been widely used for this purpose in glass and other materials [35,36]. Therefore, we do not want to study in depth the optical properties of the nanocones or optimize their dimensions, but rather we wish to compare their performance with the rest of the nanostructures and then extract conclusions. Furthermore, they will also be used as packaging for a solar cell in order to compare their performance, which will be presented in Section 3.4. Figure 6 shows the AFM image obtained from the 2D ordered nanostructures fabricated on glass as well as the height profile. The 2D nanocones obtained after RIE had a diameter of ≈230 nm and a period of 400 nm, with a depth of ≈200 nm. Since it is known that the presence of nanocones in both sides of the glass enhances considerably the *T*, we fabricated two samples, one with nanocones on a single side of the glass (sample “2D_order_1F”) and a second one with the same nanostructures on both sides of the glass (sample “2D_order_2F”).

**Figure 7:**
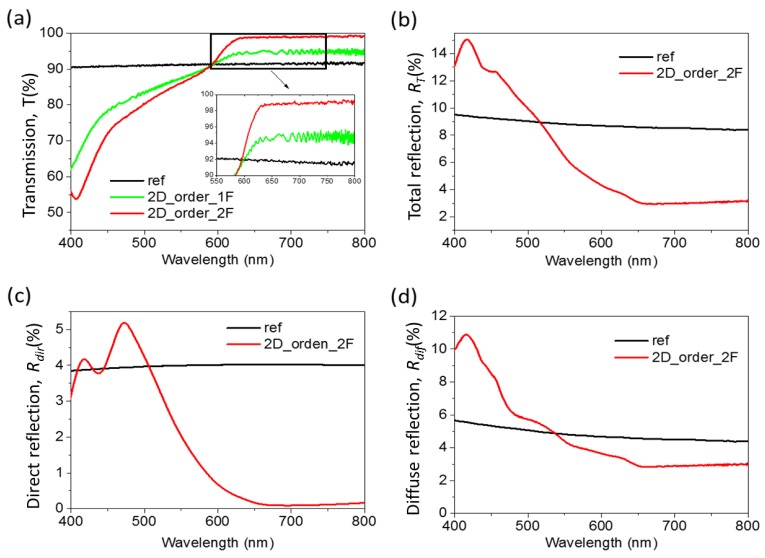
Optical measurements of ordered 2D nanostructures. (**a**) Transmission spectra (*T*) for glasses with nanostructures on a single side and on two sides. (**b**) Total reflection spectra (*R_T_*) for glasses with nanostructures on two sides. (**c**) Direct reflection (*R_dir_*) for glasses with nanostructures on two sides. (**d**) Diffuse reflection (*R_dif_*) for glasses with nanostructures on two sides.

Figure 7a shows the transmission spectra obtained for both samples. For both samples and for wavelengths exceeding 600 nm, we obtained an increase in *T* with respect to the reference sample. For the single-side, the increase was around 2%, while for the double-side, the increment was around 7%, i.e., with the nanostructuration of the two sides, we reached 99% of *T* versus 92% of the reference sample. It was also observed that for wavelengths below 600 nm, a rapid drop in transmission was seen for both samples. This has been observed previously and can be attributed to the onset of the internal reflection that occurs on ordered nanostructured surfaces, which are therefore highly dependent on the period used [37]. This effect is increased when the two air-glass-air interfaces have an index-matching, due to a higher number of internal reflections. 

Figure 7b shows the *R_T_* spectrum of the double-sided sample. This spectrum shows a high *R_T_* for wavelengths below 600 nm. However, for longer wavelengths, we obtained *R_T_* of ≈7% less than the reference sample. This agrees well with the increase in *T* obtained for these wavelengths. Figure 7c shows the *R_dir_* obtained for this glass. In this graph, it was observed that the *R_dir_* is high for short wavelengths. However, only at wavelengths between 400 and 500 nm is the *R_dir_* obtained higher than in the reference sample. This indicates, as is shown in Figure 7d, that the decrease observed in *T* is mainly due to the increase in scattering below 600 nm. 

The results obtained for ordered and disordered nanostructures with different dimensionality support the recent idea of incorporating hierarchical nanostructuration [38]. Ordering is necessary to reduce the random scattering, at least in the range of sizes that we have used for the nanostructuration. Different sizes of nanostructures are also needed in order to achieve broadband enhanced transmission. However, all of this should also entail low-cost, ease of fabrication, and demonstrated performance in a large area with sufficient mechanical robustness.

### 3.4. Solar Efficiency

**Figure 8:**
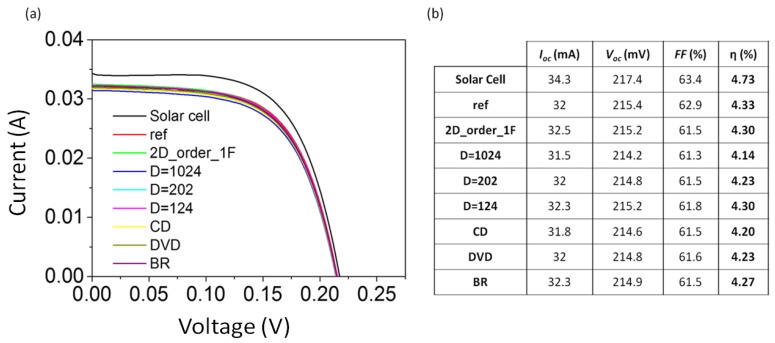
(**a**) *I*-*V* curves recorded for a solar cell with different nanostructured cover glasses (2D_ordered_2F, D = 1024, D = 202, D = 124, CD, DVD and BR), with a conventional cover glass (ref), and without cover glass (solar cell) in a solar simulator. (**b**) Values of fill factor (*FF*) and solar efficiency (η) obtained from *I*-*V* curves.

A solar simulator was used to study the behavior of nanostructured glass in a solar cell. Measurements were taken by placing the glass over a solar cell. The set was illuminated with a lamp AM1.5D simulating the solar spectrum. A solar cell of SiGeSn of 1 cm^2^ was used for the measurements. Figure 8a shows the *I*-*V* curves obtained for each of the nanostructured glasses, as well as the reference sample and the *I*-*V* curve of the solar cell without any glass. Figure 8b shows the results obtained from the *I*-*V* curves. In the filling factor (*FF*) defined as *FF* = (*I*_mp_ × *V*_mp_)/(*I*_sc_ × *V*_oc_) where *I*_mp_ and *V*_mp_ are the current and voltage at the maximum power point respectively, *I*_sc_ is the short-circuit current at zero voltage and *V*_oc_ is the open-circuit voltage at zero current. The solar efficiency was calculated as η = (*I*_sc_ × *V*_oc_ × *FF*)/(*G* × *Area*) where *G* is the power of the lamp, in this case 1 sun, and *Area* is the area of the solar cell, in our case 1 cm^2^.

**Figure 9:**
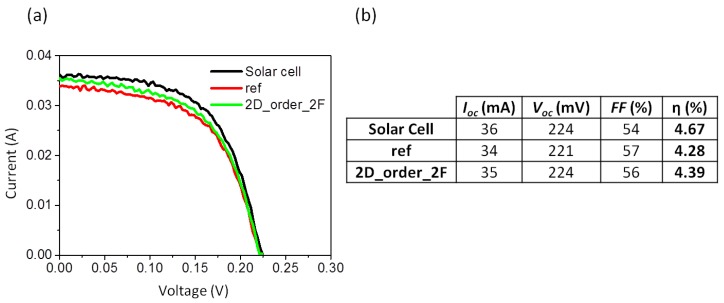
(**a**) *I*-*V* curves recorded for a solar cell with a conventional cover glass (ref), without cover glass (solar cell) and with a nanostructured cover glass (2D_ordered_2F) in a solar simulator. (**b**) Values of fill factor (*FF*) and solar efficiency (η) obtained from *I*-*V* curves.

The values obtained for *FF* and η indicate that the nanostructured glasses 2D_order_1F and D = 124 produced a better performance with efficiencies around 4.30%, which is very similar to the reference sample (η = 4.33%). The samples with the highest η are also those with the lower reflection for large wavelengths. It should be noted that η does not improve, although *T* improves, which is related to the low-efficiency of the solar cell used. Nevertheless, and as mentioned before, our study is not focused on obtaining record values but rather on the combination of clearly defined effects and the use of large-area, low-cost methods to enhance the properties of solar glass. In any case, the efficiency of the double-sided nanostructured sample, that was measured using a cell with similar characteristics to those that were used for the measurement of the remaining samples, was clearly improved. Figure 9a shows the *I*-*V* curves obtained for the solar cell without glass, with glass (reference) and for the double-sided nanostructured sample. Figure 9b shows the results for the calculation of the *FF* and η. In this case, we obtained an improvement of η to 4.39%, 0.11% higher than the reference sample. This value is clearly measured within an experimental error (which is below 0.1%). Despite the improvement seeming small, it is a significant change relative to the small efficiency of the solar cell used. Currently, record efficiency of 26.6% [39] has been achieved in solar cell panels (i.e., cells under solar glass) using silicon-based solar cells, which would result in an increment close to 0.7% if our glass was used. For multiple-junction solar cells, efficiencies can reach 46% [40] and the enhancement using our glass would be around 1.2%, which is a significant value in the field of solar energy. Therefore, we consider our results as very promising, although it is clear that more research is needed to achieve higher increments.

### 3.5. Wettability

**Figure 10:**
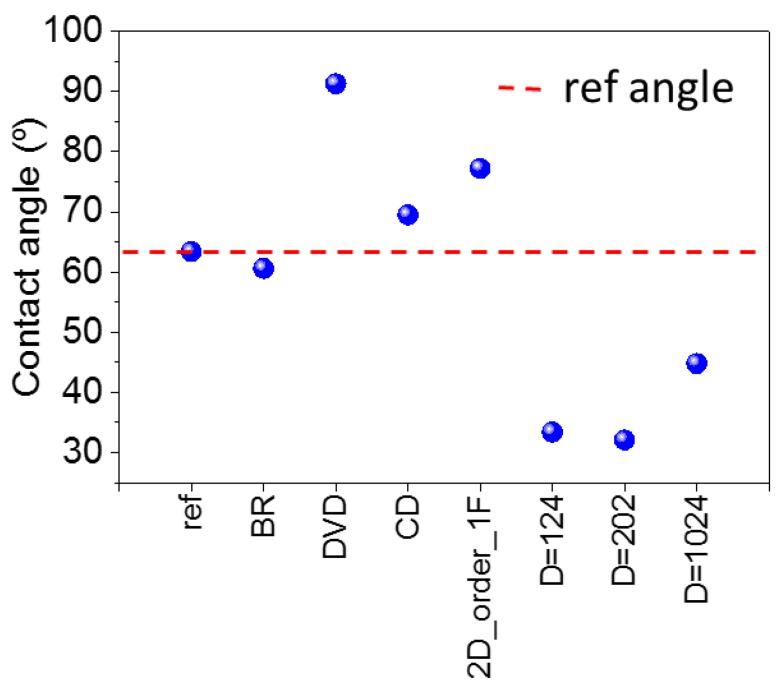
Contact angles of nanostructured glasses and reference sample.

An important property of the glasses used in solar cells is their wettability [13,21,36,41]. To understand the effect that the fabricated nanostructures could have on wettability, measurements of contact angle were carried out. A higher contact angle increases the hydrophobicity and therefore the self-cleaning capacity. Likewise, a higher hydrophilic surface increases the capacity to prevent fogging or hazing. To carry out these measurements, a drop of 0.5 μL was dropped with the help of an injecting needle and the angle of the resultant drop was measured on the glasses. Figure 10 shows the contact angles obtained for the different nanostructured glasses as well as the reference sample. No chemical treatment of the surface was undertaken. The red dotted line in Figure 10 marks the contact angle of the reference sample for a quick view of the increase or decrease in the contact angle of the material. It is observed that the 1D and 2D ordered nanostructures (with the exception of BR) increased the hydrophobicity of the material. The values obtained were 91.3° for DVD and 77.2° for 2D_order_1F, while the reference sample showed a value of 63.4°. In contrast, the fabrication of 2D disordered nanostructures on glass decreased the contact angle, and angles between 45° and 30° were obtained. These results clearly show that the manufacture of ordered nanostructures produces an increase in hydrophobicity, and may improve the self-cleaning ability of the material. They also show that the manufacture of disordered nanostructures increases the hydrophilic capacity, which may also improve the ability to prevent fog formation in the glass. In addition, we can conclude that it is possible to add, by selecting the appropriate nanopattern, wettability properties (hydrophobic or hydrophilic) without significantly decreasing the efficiency of the solar cell. 

## 4. Conclusions

We have studied the effect on the optical and wettability properties of the fabrication of nanostructured solar glass using different size, order and dimension. The fabrications of the nanostructures were based on soft lithography and colloidal lithography, both low-cost techniques that permit the nanopatterning of large areas. The results support that ordering is necessary to reduce the random scattering and that different sizes of nanostructures are also required to achieve broadband enhanced transmission. An increase in the transmission of solar glass for 1D and 2D ordered nanostructures was obtained with a maximum value of 99% when 2D ordered nanostructures were fabricated on the two sides of the solar glass. Using it, we obtained an increase in the solar cell efficiency of 0.11% with respect to the reference glass. This enhancement could be of more than 1% using high-efficiency solar cells such as multi-junction solar cells. Contact-angle measurements showed that the fabrication of ordered nanostructures on solar glass can increase the contact angle. On the other hand, a decrease in the contact angle was observed when 2D disordered nanostructures were fabricated, which may improve the anti-fogging capacity. The wettability properties can be improved by selecting the adequate nanopattern without decreasing the efficiency of the solar cell. However, all of this should also entail very low-cost, ease of fabrication and demonstrated performance in a large area with sufficient mechanical robustness.
